# Genomic characterization of *Bacillus cereus* sensu stricto 3A ES isolated from eye shadow cosmetic products

**DOI:** 10.1186/s12866-022-02652-5

**Published:** 2022-10-05

**Authors:** Nadine Yossa, Rebecca Bell, Sandra Tallent, Eric Brown, Rachel Binet, Thomas Hammack

**Affiliations:** Office of Regulatory Science, FDA, College Park, MD 20740 USA

**Keywords:** Complete genome sequence, *Bacillus cereus*, Virulence factors, Resistant genes, Cosmetics

## Abstract

**Background:**

The *Bacillus cereus* group, also known as *B. cereus* sensu lato (s.l.) contains ubiquitous spore-forming bacteria found in the environment including strains from the *B. cereus* sensu stricto (s.s.) species. They occur naturally in a wide range of raw materials and in consumer products. Characterizing isolates that have survived in consumer products allows us to better understand the mechanisms that permit spores to persist and potentially cause illness. Here we characterize the draft genome sequence of *B. cereus* s. s. 3A-ES, originally isolated from eye shadow and since investigated in several cosmetic studies and compared it to other top ten published complete genome sequences of *B. cereus s.l*. members.

**Results:**

The draft genome sequence of *B. cereus s.s.* 3A ES consisted of an average of 90 contigs comprising approximately 5,335,727 bp and a GC content of 34,988%, and with 5509 predicted coding sequences. Based on the annotation statistics and comparison to other genomes within the same species archived in the Pathosystems Resource Integration Center (PATRIC), this genome “was of good quality. Annotation of *B. cereus s.s.* 3A ES revealed a variety of subsystem features, virulence factors and antibiotic resistant genes. The phylogenetic analysis of ten *B. cereus* group members showed *B. cereus* s.s. 3A-ES to be a closely related homolog of *B. cereus* s.s. ATCC 14,579, an established reference strain that is not adapted for cosmetic microbiological studies. Survival of 3A-ES in eye shadow could be linked to predicted stress-response genes and strengthened by additional stress-response genes such as VanB-type, VanRB, CAT15/16, BcrA, BcrB, Lsa(B), and recA that are lacking in *B. cereus* s.s. ATCC 14,579.

**Conclusion:**

Our genomic analysis of *B. cereus s.s.* 3A-ES revealed genes, which may allow this bacterium to withstand the action of preservatives and inhibitors in cosmetics, as well as virulence factors that could contribute to its pathogenicity. Having a well-characterized strain obtained from eye-shadow may be useful for establishing a reference strain for cosmetics testing.

## Introduction

Microbes in the *Bacillus cereus* group [or *B. cereus* sensu lato*; s.l.*] are spore-forming organisms ubiquitous in the environment. As such, it is possible for *B. cereus* s.l. to contaminate a wide range of consumer products, including cosmetics, either as raw materials or during manufacture [1]. *B. cereus s.l.* contamination of cosmetics used near the eyes [2, 3] can result in eye infections, particularly among people who have existing trauma or damage in this area [4, 5]. Some of those infections can be serious enough to cause vision loss [6]. Any pathogen that is capable of being cultured from cosmetic products will most likely carry genes for persistence in cosmetics matrices and for withstanding the preservatives that are intended to prevent the growth of bacteria during normal consumer use of the product. Therefore, by characterizing isolates found as contaminants in the products, we can identify factors which may contribute to pathogenicity and persistence, as well as identify isolates that could become useful reference strains.

Genomic analyses can identify which toxin-producing capabilities a given isolate carries, and helps researchers better understand whether other virulence genes were acquired [7]. The *B. cereus s.l.* group consists of at least twelve closely related species: *B. anthracis*, *B. cereus s.s.*, *B. thuringiensis*, *B. mycoides*, *B. pseudomycoides*, *B. weihenstephanensis*, *B. cytotoxicus*, *B. wiedmanni*, and *B. toyonensis*, and the recently identified *B. paranthracis, B. pacificus, B. tropicus, B. albus, B. mobilis, B. Luti, B. proteolyticus, B. nitratireducens, B. paramycoides, gaemokensis, B. manliponensis, B. bingmayongensis,* and *B. fungorum* [8]. Some carry potent toxins [9] for example, the deadly effects of *B*. *anthracis,* the etiological agent of anthrax, are due to two large plasmids, pXO1 and pXO2 which produce the tripartite anthrax toxin [7]. The insect pathogen *B. thuringiensis* produces crystal toxins that make it useful as a commercial biopesticide [10], although this organism can also produce immune inhibitor metalloproteases capable of causing significant eye damage [11]. *B. cereus s.s.* can produce toxins causing gastrointestinal diseases, wound or systemic infections, and eye infections [6, 8, 12–14].

The persistence of *B. cereus s.l.* in cosmetic matrices may be in part due to the resilience of their spores, which can withstand harsh environments; however, some strains may also carry specific genes which confer to resistance to antimicrobials or preservatives. To meet the U.S. FDA Bacteriological Analytical Manual (FDA BAM) guidelines for cosmetics, preservatives are used to prevent the persistence of high-virulence microbial pathogens and keep the total number of aerobic microorganisms per gram low [15]. *B. cereus s.l.* strains which persist despite preservatives pose hygienic challenges and therefore may be good microbial candidates for cosmetics safety research. In the past, we observed that *B. cereus s.s.* reference strain ATCC14579 was not fully adapted for long term survival in eye cream preserved with parabens [16]. Here, we genetically characterize a strain of *B. cereus* s.s, designated “3A-ES”, originally described as *B. cereus* 3A, isolated from eye shadow in 2014 as part of a microbial investigation of eye area cosmetics formulated using nontraditional preservatives [17]. The investigated cosmetics were purchased from ordinary retail stock and had been formulated using organic powders of tapioca, corn, organic seed oils, minerals, mica and iron oxides, and plant extracts (thyme, tea tree, rosemary, and sweet orange oil), some of which could have been intended to serve as antibacterial components. The “traditional” preservative systems more typically used for cosmetic products consist of organic acids, alcohols and phenols, aldehydes and formaldehyde releasers, isothiazolinines, biguanides, or quaternarium ammonium compounds [18]; such ingredients were not present in these cosmetics [17].

By sequencing and analyzing the 3A-ES genome, we expect to identify genes associated with persistence and pathogenesis in order to better understand the survival of this pathogen in cosmetics. This may also contribute to ongoing discussions of *B. cereus s.l.* phylogenies [14]. The completed *B. cereus s.s.* 3A-ES genome has been submitted to Genbank under accession number JAEPEY000000000.®

## Materials and methods

### Obtaining 3A-ES from naturally contaminated cosmetics samples

Our laboratory received an analytical report of isolation of *B. cereus* s.s. 3A-ES from cosmetic samples*.* 3A-ES was first identified in June 2014 by FDA in cosmetics samples randomly collected from retail store shelves between 4/28/2014 and 4/29/2014. The eye cosmetic samples were packaged in a cardboard box, and consisted of 15 plastic pot type containers, each holding 0.5 oz of eye colors.

The samples were described on the report as analyzed following FDA BAM Chapter 23 for creams and oil-based products. Briefly, 1-g samples were aseptically added into 20 × 150 mm screw-cap tubes containing 1 ml sterile Tween® 80 plus five to seven 5-mm glass beads, homogenized with vortex mixer and adjusted to 10 ml total volume with 8 ml sterile Modified Letheen Broth (MLB; BAM media M79) to make the 10^–1^ dilution samples. Five additional decimal serial dilutions tubes were prepared by adding 5 ml of previous dilution to 45 ml of sterile MLB. Bacterial counts were determined after plating 0.1 ml of appropriate dilution onto duplicate Modified Letheen agar plates (MLA; BAM media M78) and incubation for 72 h at 30 °C. Morphologically dissimilar colony types were streaked out in parallel onto MLA and onto MacConkey agar plates that are inhibitory for Gram-positive bacteria. The report revealed that biochemical testing was performed to delineate the organisms to the species level,

*Bacillus* strains were further characterized with the VITEK® 2 automated microbiology system (bioMerieux, Durham, NC) using a BCL (Gram-positive spore-forming bacilli) reagent card, following manufacturer’s recommendations. Identification results were reported as correct identification to a single species, or to *B. cereus s.s.*, *B. thuringiensis* and *B. mycoides* in a slashline indicating that the biopattern was insufficient to discriminate between those species, or low discrimination indicating that supplementary tests were needed for discrimination, or unidentified [18]. Upon receiving the bacterial stab culture vials, a small amount was taken with a sterile loop and streaked in quadrant on TSA plates. Then, an isolated colony was taken with a loop, inoculated in Nutrient Broth (NB; Difco, USA) and cultured for 24 h at 35º C with agitation. Then, after homogenization, 700 µl of the culture suspension was aliquoted into 2 ml sterile cryovials containing 300 µl of 50% of sterile glycerol.These vials were sealed, labeled as “3A”, and stored at -80 ºC for future use [19]. *B. cereus s.s.* colonies were then confirmed onto *Bacillus cereus* rapid agar (BACARA®) (bioMerieux, Durham, NC) where they grow as pink colonies with an orangey halo [18]. Further investigations of the strain labeled as “3A” presented no rhizoid growth or protein crystals. “-ES” was later added to the name of that strain to reflect its origin (eye shadow).

### Whole genome sequencing

Genomic DNA was extracted after overnight incubation at 35 °C in NB using the DNeasy Blood and Tissue Kit (Qiagen Inc, Valencia, CA). DNA concentrations were measured using a Qubit 3.0 fluorometer (Life Technologies, MD). Sequencing libraries were prepared according to Nextera XT protocols using 0.2 ng/μl of DNA and sequenced on the Illumina MiSeq desktop sequencer (Illumina, San Diego, CA) using MiSeq Reagent V2 kits (500 cycles of paired end reads) following the manufacturer’s guidelines. Sequences were trimmed using the Illumina software using standard parameters. The resulting trimmed Fastq data sets were de novo assembled using Unicycler v0.4.8 with default parameters. Assembled genomic data was submitted to PATRIC and Rapid Annotation using Subsystem Technology tool kit (RASTtk) [20, 21] for annotation and comparative analysis, to predict the presence of genes relevant to risk assessment (virulence factors, antibiotic resistance genes, drug targets, and human homologs).

### Analyses for virulence factors, AMR genes and other genes relevant to risk assessment

Genes relevant to risk assessment include virulence factors, antibiotic resistance genes, drug targets (proteins that are targeted by known, approved, or experimental small molecule drugs), and human homologs. For each class, reference genes are selected from reputable external databases, or manually curated by the PATRIC team, then mapped using BLASTP to their homologs in newly submitted genomes, based on sequence similarity.

The PATRIC service integrates and maps virulence factor genes from the following sources: the Virulence Factor Database (VFDB) [22], the Transporter Classification Database (TCDB) [23], and the PAThosystems Resource Integration Virulence Factors Center (PATRIC_VF) [24]. Known antibiotic resistance genes were integrated and mapped from the Comprehensive Antibiotic Resistance Database (CARD), and the National Database of Antibiotic Resistant Organisms (NDARO) [25], then compared with genes found in the sequence of 3A-ES. Additionally, PATRIC analyzes genomes for their number of subsystems that each represents genes involved in a specific biological process.

### Phylogeny and comparative genomic analyses to closely related *B. cereus s.l.* isolates

To create the phylogenetic dendrogram, the National Center for Biotechnology Information (NCBI) staff manually select and categorize high-quality reference genomes. PATRIC provides reference databases and other representative genomes and includes these in the phylogenetic analysis as part of their Comprehensive Genome Analysis reports. The ten genomes most closely related to *B. cereus s.s.* 3A-ES were identified by Mash/MinHash [26], and PATRIC global protein families (PGFarms) [27]. The protein sequences from those families were aligned with MUSCLE [28], and the nucleotides coding gene sequences were aligned using the Codon align function of BioPython [29]. A concatenated alignment of all proteins and nucleotides were writen to a phylip formatted file, and then a partitions file for RaxML [30], was generated, describing the alignment in terms of the proteins and then first, second and third codon positions. Support values were generated using 100 rounds of the Rapid bootstrapping option [31].

## Results and discussion

### Whole genome sequencing of *B. cereus s.s.* 3A ES

We submitted the assembled draft genome of *B. cereus s.s.* 3A ES to the comprehensive genome analysis service at PATRIC [21]. Based on the annotation statistics and compared to other *B. cereus s.s.* genomes in PATRIC, our *B. cereus s.s.* 3A ES genome was determined to be of good quality. The assembly and annotation statistics are displayed in Table 1.

**Table 1:** Assembly and annotation statistics of *B. cereus s.s.* 3A ES

Genome accession number JAEPEY000000000	
Coarse consistency (%)	99.8
Fine consistency (%)	98.2
Completeness (%)	100
Contamination (%)	0
Contig count	90
GC Content	34.9880
DNA size (bp)	5,335,727
Contigs N50 (bp)	192,359
Contigs L50	9
Overpresent Roles	17
Underpresent Roles	7
Predicted Roles	1370
Completeness Roles	43
Total Distinct Roles	3308
Protein-Encoding Genes with Functional Assignment	3408
Protein-Encoding Genes without Functional Assignment	2101
% Protein-Encoding Feature Coverage	103.25
% Features that are Hypothetical	38.14
% Features that are in Local Protein Families	96.57
Number of subsytems	287
Number of coding sequences (CDS)	5509
Number of tRNA	60
Number of rRNA	4
Plasmid	0

### General genomic annotation

**Fig. 1:**
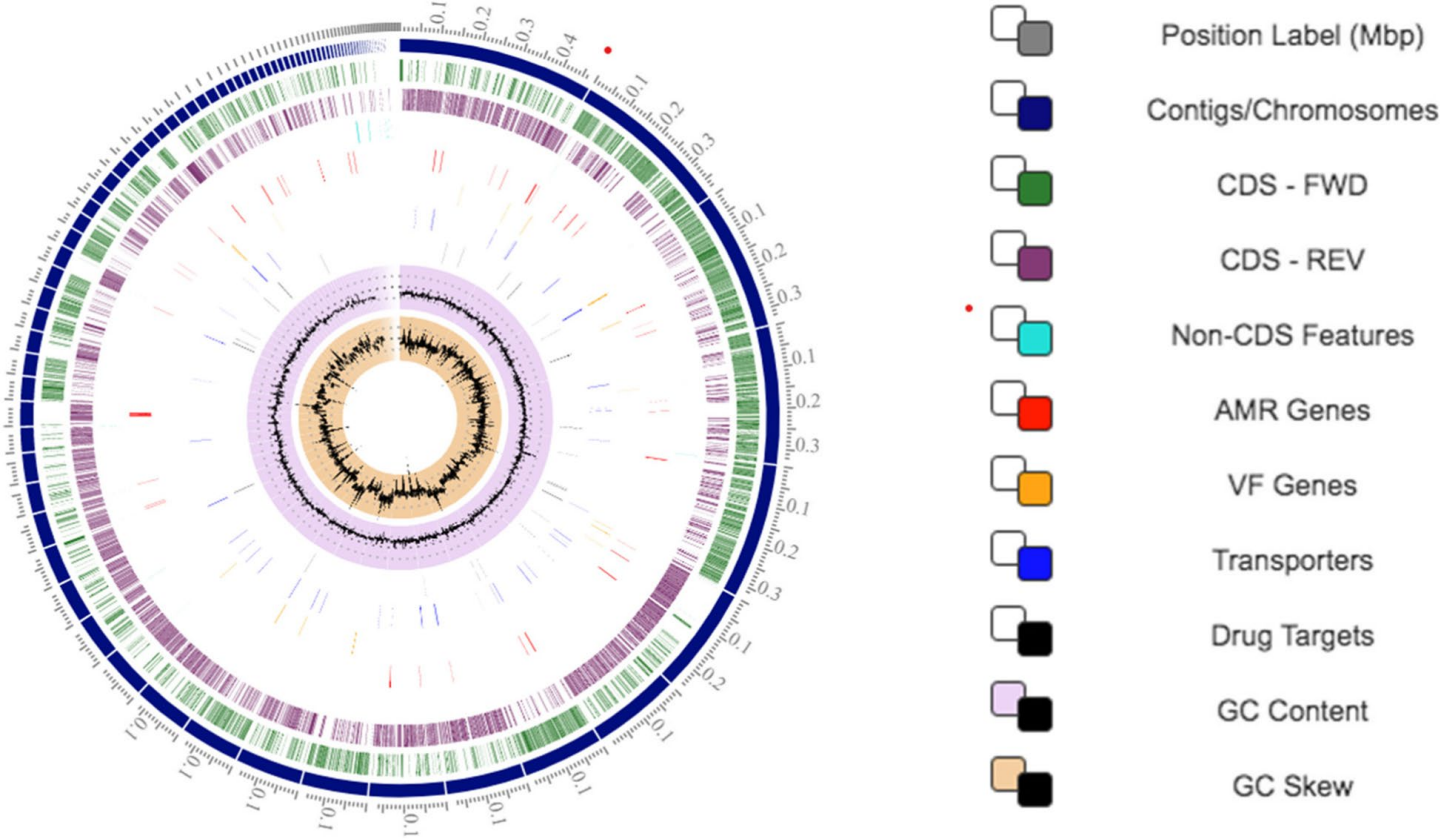
Circular display of the distribution of the draft genome annotations in *B. cereus s.s.* 3A ES. From the outer to the inner rings, this display includes: the contigs, coding sequences (CDS) on the forward (FWD) strand, CDS on the reverse (REV) strand, RNA genes, CDS with homology to known antimicrobial resistance genes, CDS with homology to known virulence factors, GC content and GC skew. The colors of the CDS on the forward and reverse strand should have indicated to which subsystem (see Fig. 2) these genes belong

Figure 1 provides a circular display of the distribution of the draft genome annotations for *B. cereus s.s.* 3A ES. 5,509 protein coding sequences (CDS), 60 transfer RNA (tRNA), and 4 ribosomal RNA (rRNA) genes were identified.

**Fig. 2:**
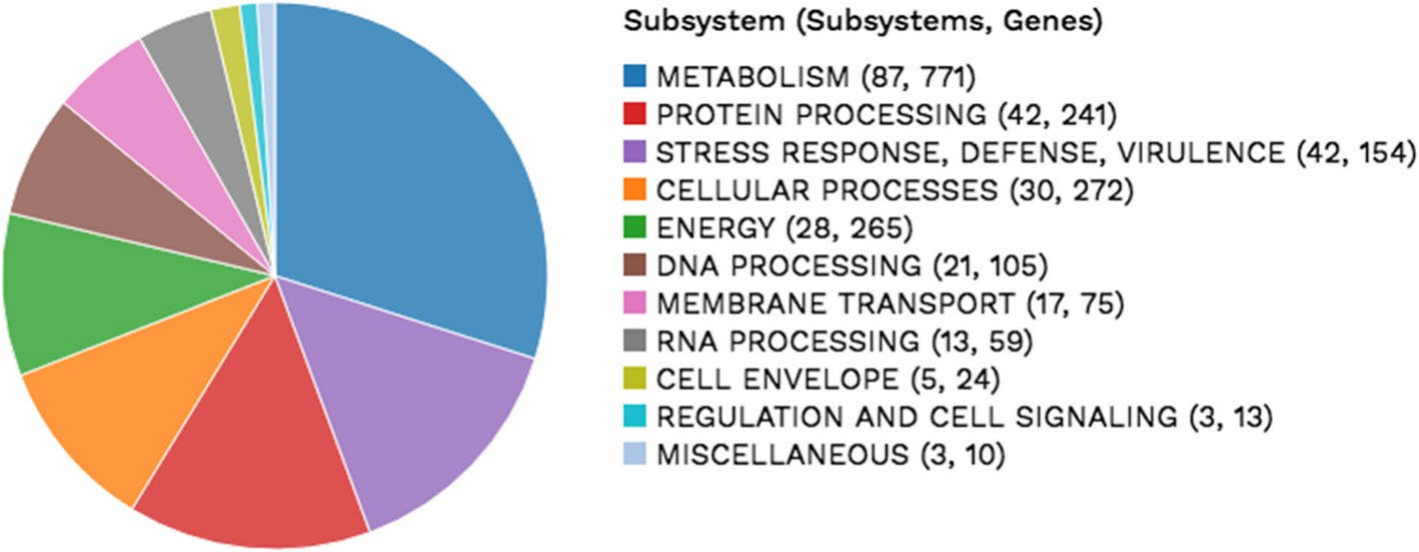
Overview of subsystems within the *B. cereus s.s.* 3A-ES genome

Within these coding sequences are subsystems, which are sets of proteins that together implement a specific biological process or structural complex [32]. We identified 287 subsystems in the *B. cereus s.s.* 3A-ES genome, of which the largest number were devoted to metabolic processes, followed by protein processing, virulence, stress response, and defense (Fig. 2).

### Virulence factors, AMR genes and other genes relevant to risk assessment

Our analyses annotated genes in the *B. cereus s.s.* 3A-ES genome associated with virulence, stress response, and defense functions. Many of these genes are homologous to known genes which function as transporters, virulence factors, drug targets, and provide resistance to a range of antibiotics. Table 2 lists these genes, the number of genes found within each category, and the specific source database where the homologies were found.

**Table 2:** Genes relevant to risk assessment in *B. cereus s.s.* 3A-ES

Gene category	Genes	Source
Virulence Factor	12	Victors
Virulence Factor	9	VFDB
Transporter	49	TCDB
Drug Target	27	Drug Bank
Antibiotic Resistance	54	PATRIC
Antibiotic Resistance	7	CARD
Antibiotic Resistance	5	NDARO

*VFDB* virulence factor database, *TCDB* transporter classification database, *PATRIC* PAThosystems Resource Integration Center, *CARD*, comprehensive antibiotic resistance database, *NDARO*: National Data of Antibiotic Resistant Organisms

Some of the more important virulence genes, selected among the genes relevant to risk assessment causing gastrointestinal and non-gastrointestinal diseases were found in *B cereus* 3A-ES and are presented in Table 3, along with some of the other organisms which also carry these genes.

**Table 3:** Source and virulence genes found in *B. cereus s.s.* 3A-ES

Source ID	Source Organism	Gene	Product	Subject Coverage	Query Coverage	Identity	E-value
VFG016223	BC ATCC 10987	*cytK*	Cytolytic pore-forming protein = > Cytotoxin K	100	100	97	1e-192
VFG016270	BC ATCC 10987	*nheA*	Non-hemolytic enterotoxin A	100	100	96	1e-209
VFG016338	BA str. Sterne	*inhA*	Immune inhibitor A, metalloprotease (EC 3.4.24.-)	100	100	95	0.0
VFG016263	BC ATCC 14579	*hblC*	Hypothetical protein	100	100	96	1e-238
VFG016278	BC ATCC 10987	*nheB*	Non-hemolytic enterotoxin lytic component L1	100	100	99	1e-225
VFG016254	BC ATCC 14579	*hblA*	Hypothetical protein	100	100	98	1e-269
VFG016216	BA str. Sterne	*BAS3109*	Thiol-activated cytolysin	100	100	96	1e-289
VFG016260	BC ATCC 14579	*hblD*	Hypothetical protein	100	100	99	1e-231
Drugbank	BC	*plc*	Broad-substrate range phospholipase C (EC 3.1.4.3)	100	100	95	1e-164 1e-164
VFG016286	BC ATCC 10987	*nheC*	Enterotoxin C	100	100	94	1e-194

VFDB: virulence factor database; BC: *B. cereus s.s.*; BA: *B. anthracis*; Victors: virulence factors mapped from Victors; Query coverage: a number that describes how much of the query sequence is covered by the target sequence. Identity: a number that describes how similar the query sequence is to the target sequence. E-value: the Expected Value describes how many times you would expect a match to occur by chance in a database of this size.

Many of the virulence factors secreted by members of the *B. cereus s.s.* group, including enterotoxins, hemolysins, phospholipases and proteases are activated by the pleiotropic transcriptional regulator PlcR (phospholipase C regulator) [33]. The activity of PlcR peaks at the onset of the stationary growth phase and is dependent upon the presence of PapR, a small signaling peptide that acts as a quorum-sensing effector [34]. Specifically, PapR is exported and subsequently reimported into the bacterial cell as a processed heptapeptide which then interacts with PlcR to facilitate binding to the nucleotidic sequence PlcR box, which is located upstream of its target genes [35]. This pairing of PlcR/PapR transcription regulators were found in the genome of *B. cereus s.s.* 3A-ES, and the presence of PlcR has previously been associated with the rapid destruction of retinal function in cases of *Bacillus* endophalmitis [36].

In addition to these transcription regulators, genes for toxins and enzymes common to *B. cereus s.s.* group members were also found in *B. cereus s.s.* 3A-ES. These genes included three nonhemolytic enterotoxin genes (*nheABC*), the hemolytic enterotoxin genes (*hblCDA*) and the gene encoding cytotoxin K (*cytK*), which is responsible for gastrointestinal infections. Pore-forming toxins were also present, including thiol-activated cytolysins, hemolysin A, and hemolysin III, that play roles in non-gastrointestinal infections [37]. *B. cereus s.s.* 3A-ES also carries genes for enzymes such as phospholipase C and collagenases. Beecher, et al. (2000) demonstrated how tripartite hemolysin BL, phosphatidylcholine-phospholipase C, and collagenase could contribute to the severity of *B. cereus s.s.* endophthalmitis [38]. Another notable toxin category found in 3A-ES was immune inhibitor A (InhA)-type metalloproteases. These have been shown to allow spores of *B. cereus s.s.* to survive and escape macrophage attacks [39], and more recently Inh-A metalloproteases have been shown to be independently associated with both retinal damage and deterioration of the vitreous portion of eyes infected with *Bacillus* species [11].

Although these toxins are closely associated with tissue-destructive/reactive exoenzyme production [12], based on the analysis of strains which carry different arrangements of these genes, damage to eye tissues may not be solely attributable to individual toxins alone. Virulence might result from combinations of toxins or because of toxins in synergy with other factors [40–42].

### Antimicrobial resistance genes

We identified antimicrobial resistance (AMR) genes carried by *B. cereus s.s.* 3A-ES using PATRIC’s curated collection of representative AMR gene sequence variants and a k-mer based AMR gene detection method [21]. One of the most interesting findings was the potential presence of sigma factor B, which, when activated by stress conditions (including heat or acid shock, high osmolarity, high ethanol concentrations, high or low pH, sodium chloride, and/or oxidizing agents) can trigger transcription of genes conferring stress resistance to vegetative cells [43]. Studies have indicated that sigma B also plays an important role in a wide range of other protective mechanisms, such as antibiotic resistance, pathogenesis and cellular differentiation processes, biofilm formation and sporulation [44, 45].

Based on the PATRIC comparison, several other compounds related to antibiotic resistance and toxin production were predicted to be in *B. cereus s.s.* 3A-ES genome (Table 4). Specifically, we identified genes conferring resistance to fosfomycin, daptomycin, vancomycin and teicoplanin, bacitracin, ciprofloxacin, chloramphenicol, tetracycline, mupirocin, beta-lactamases ambler class A and B, fusidic acid, macrolides, lincosamides, treptogramins, ketolides, and oxazolidinones (MLSKO). Further, we found genes which are likely to support survival of 3A-ES in the presence of antimicrobial agents, some of which are used as traditional preservatives in cosmetics, including triclosan and trimethoprim chromium compounds, and the chemical element, arsenic. The use of non-traditional preservatives in the cosmetic product it was isolated from might have accentuated the resistance hence the survival of 3A-ES in many types of cosmetic products. Further characterization of 3A-ES, using direct challenges to cultured cells, could confirm if this range of resistance occurs in vivo.

**Table 4:** Stress response defense virulence in *B. cereus s.s.* 3A ES

Subclass	Subsystem Name	Gene Count	Role Count	Active
Toxins and superantigens	Pore-forming cytolytic toxins	1	1	active
Stress Response: Osmotic stress	Choline uptake and conversion to betaine clusters	8	5	active
Stress Response: Osmotic stress	Osmoregulation	2	2	active
Stress Response: Heat/cold shock	Heat shock dnaK gene cluster extended	16	16	active
Stress Response: Heat/cold shock	Cold shock proteins of CSP family	6	2	active
Stress Response: Electrophile toxicity	Bacillithiol synthesis	4	3	active
Stress Response	Cluster containing Glutathione synthetase	2	2	likely
Stress Response	Glutathione: Non-redox reactions	4	2	likely
Stress Response	CoA disulfide thiol-disulfide redox system	1	2	active
Stress Response	Protection from Reactive Oxygen Species	9	7	active
Stress Response	Universal stress protein family	2	1	active
Stress Response	Repair of Iron Centers	1	1	active
Stress Response	Glutathione: Redox cycle	1	1	active
Stress Response	Stress proteins YciF, YciE	1	1	active
Resistance to antibiotics and toxic compounds	Aminoglycoside modifying enzymes: O-nucleotidyltransferases	1	1	active
Resistance to antibiotics and toxic compounds	Fosfomycin resistance	1	1	active
Resistance to antibiotics and toxic compounds	Resistance to Daptomycin	14	7	active
Resistance to antibiotics and toxic compounds	Resistance to Vancomycin and Teicoplanin	7	3	likely
Resistance to antibiotics and toxic compounds	Beta-lactamases Ambler class A	2	1	active
Resistance to antibiotics and toxic compounds	Antibiotic targets in protein synthesis	9	8	active
Resistance to antibiotics and toxic compounds	Antibiotic targets in metabolic pathways	7	5	active
Resistance to antibiotics and toxic compounds	Antibiotic targets in DNA processing	4	4	active
Resistance to antibiotics and toxic compounds	VraTSR and LiaFSR three-component regulatory systems	5	5	active
Resistance to antibiotics and toxic compounds	Copper homeostasis: copper tolerance	1	1	active
Resistance to antibiotics and toxic compounds	Fusidic acid resistance	2	2	likely
Resistance to antibiotics and toxic compounds	Macrolides, lincosamides, streptogramins, ketolides, oxazolidinones (MLSKO) resistance: enzymatic degradation	1	1	active
Resistance to antibiotics and toxic compounds	Bacitracin resistance	3	3	active
Resistance to antibiotics and toxic compounds	Antibiotic targets in cell wall biosynthesis	6	3	active
Resistance to antibiotics and toxic compounds	Vancomycin resistance, D-Ala-D-Ala dipeptidases and carboxypeptidases	1	1	active
Resistance to antibiotics and toxic compounds	Arsenic resistance	7	5	active
Resistance to antibiotics and toxic compounds	Antibiotic targets in transcription	3	3	active
Resistance to antibiotics and toxic compounds	Aminoglycoside modifying enzymes: N-acetyltransferases	1	1	likely
Resistance to antibiotics and toxic compounds	Beta-lactamases Ambler class B	1	1	active
Resistance to antibiotics and toxic compounds	Resistance to Triclosan	1	1	active
Resistance to antibiotics and toxic compounds	Resistance to chromium compounds	1	1	active
Resistance to antibiotics and toxic compounds	Mupirocin resistance	2	1	likely
Resistance to antibiotics and toxic compounds	Chloramphenicol resistance	1	1	active
Resistance to antibiotics and toxic compounds	Tetracycline resistance, all mechanisms	2	2	active
Resistance to antibiotics and toxic compounds	Macrolides, lincosamides, streptogramins, ketolides, oxazolidinones (MLSKO) resistance: ribosomal protection	1	1	active
Invasion and intracellular resistance	Listeria surface proteins: Internalin-like proteins	3	1	active
Host–pathogen interactions	Hydrolysis of sphingomyelin	2	2	active
	Hfl operon	5	5	active

The genome of *B. cereus s.s.* 3A-ES also revealed factors conferring resistance to multiple drugs, including multidrug resistance proteins, efflux pumps (MFSF, HrtAB, BcrA, BcrB, YkkCD), transcriptional regulator (BkdR), ABC-type transporter, and penicillin-binding proteins.

One of our original interests in this project was to investigate whether 3A-ES carried spore-associated features that might confer survival advantages in cosmetics matrices. In addition to the genes for InhA-type metalloproteases and sigma B, our analyses revealed genes coding multiple proteins that could contribute to spore resilience, providing resistance to oxidizing agents and chemicals by blocking toxic molecules [46]. These genes included spore coat proteins (CotB, CotO, CotW, CotX, CotY/CotZ), inner spore coat proteins (CotD, CotH), outer spore coat protein (CotE), exosporium protein B, spore coat protein (CotG), protein CotJA, polypeptide composition of the spore coat protein (CotJB), and manganese catalase spore coat protein (CotJC).

Genes for superoxide dismutase (Mn) were present in the genome. These are known to be involved in bacterial oxidative stress responses, usually generated after *B. cereus s.s.* cells are exposed to lethal or sub-lethal stresses levels of acids [47–49]. Further, superoxide dismutase has been reported to contribute to the severity of *B. cereus s.s.* endophthalmitis [42].

The wide range of stress response, defense, and resistance genes found in *B. cereus s.s.* 3A-ES suggest that it could indeed protect itself effectively from drugs, antibiotics, toxic heavy metals, low pH as well as from the action of the preservative systems used in cosmetics. In addition, non-traditional preservatives contained in the product contribute to its survivability A summary of the potential AMR genes found in 3A-ES genome and the corresponding AMR mechanisms is provided in Table 5.

**Table 5:** Antimicrobial resistance genes found in *B. cereus s.s.* 3A ES

AMR Mechanism	Genes
Antibiotic inactivation enzyme	BcII family, CatA15/A16 family, FosB
Antibiotic target in susceptible species	Alr, Ddl, dxr, EF-G, EF-Tu, folA, Dfr, folP, gyrA, gyrB, inhA, fabI, Iso-tRNA, kasA, MurA, rho, rpoB, rpoC, S10p, S12p
Antibiotic target protection protein	BcrC, Lsa(B)
Efflux pump conferring antibiotic resistance	BcrA, BcrB, YkkCD
Gene conferring resistance via absence	gidB
Protein altering cell wall charge conferring antibiotic resistance	GdpD, MprF, PgsA
Protein altering cell wall structure conferring antibiotic resistance	VanXY-unclassified
Regulator modulating expression of antibiotic resistance genes	LiaF, LiaR, LiaS, VanB-type, VanF/M-type

### Phylogenetic tree analysis

**Fig. 3:**
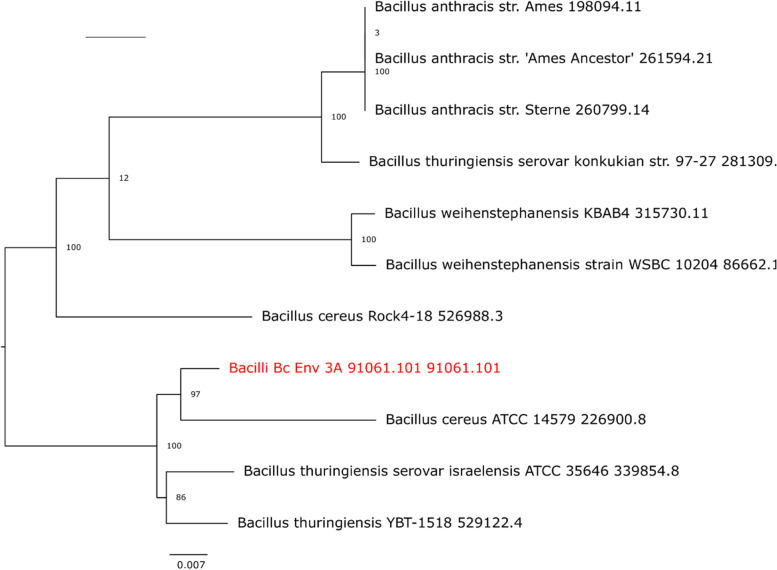
Phylogenetic tree constructed based on the homologous genomes of NCBI’ s high quality and of PATRIC’ reference and representative genomes. *B. cereus s.s*

Organisms grouped within *B. cereus s.l.* can vary widely in virulence and pathogenicity, with some *B. cereus s.s.* strains causing illnesses that more closely resemble the anthrax caused by *B. anthracis*, raising questions about *B. cereus s.s.* species categorization and nomenclature [14]. Our phylogenetic tree comparing 3A-ES to ten closely related members of *B. cereus s.l.* group, as identified by PATRIC, confirmed that *B. anthracis* and *B. cereus s.s.* 3A-ES evolved from a common ancestor, and showed the strain having the closest evolutionary relationship to 3A ES was *B. cereus s.s.* ATCC 14,579, an established reference strain, with robust support, bootstrap of 97 (Fig. 3). This value indicates that *B. cereus s.s.* 3A ES carry a set of virulence genes like those carried by ATCC 14,579. The genome of *B. cereus s.s.* ATCC 14,579 contains 28 functional PlcR boxes, forming a *plcR* regulon of at least 45 genes, of which 22 genes may be secreted in the extracellular medium, 18 genes are bound to cell wall structures (membrane or peptidoglycan layer) and 4 genes code for cytoplasmic regulators [50]. Moreover, a preliminary comparison of the genes relevant to risk assessment in PATRIC revealed that *B. cereus s.s.* 3A-ES had additional genes such as VanB-type, VanRB, CAT15/16, BcrA, BcrB, Lsa(B), and *recA* that may contribute to its higher resistance and survival in cosmetic products, compared to ATCC 14,579.

## Conclusion

Our analyses of the *B. cereus s.s.* 3A ES draft genome revealed multiple factors that may contribute to pathogenicity and resistance to stress conditions, including the presence of preservatives which are usually added to prevent bacterial survival in consumer products. This may explain why viable bacteria could be cultured from eye shadow. The ability to resist preservatives makes *B. cereus s.s.* 3A ES a good candidate to screen for in cosmetic testing. *B. cereus s.s.* 3A ES will be used in our future studies for developing methods for the detection and recovery of bacterial microorganisms in cosmetic products.

## Data Availability

The raw data supporting the conclusions of this manuscript will be made available to the authors, without undue reservation, to any qualified researcher. *B. cereus s.s.* 3A-ES whole genome project has been deposited in Genbank under accession number JAEPEY000000000. The raw Illumina data from BioProject accession number PRJNA574468 were submitted to the NCBI Sequence Read Archive (SRA) under experiment accession number SRR13386521. The raw data supporting the conclusions of this manuscript will be made available by the corresponding author, without undue reservation, to any qualified researcher.
